# Genome composition in *Brassica* interspecific hybrids affects chromosome inheritance and viability of progeny

**DOI:** 10.1007/s10577-023-09733-9

**Published:** 2023-08-19

**Authors:** Elvis Katche, Elizabeth Ihien Katche, Paula Vasquez-Teuber, Zurianti Idris, Yu-tzu Lo, David Nugent, Jun Zou, Jacqueline Batley, Annaliese S. Mason

**Affiliations:** 1 Plant Breeding Department, Justus Liebig University, Heinrich-Buff-Ring 26-32, 35392 Giessen, Germany; 2 Plant Breeding Department, University of Bonn, Katzenburgweg 5, 53115 Bonn, Germany; 3 School of Agriculture and Food Sciences, The University of Queensland, Brisbane, 4072 Australia; 4 Department of Plant Production, Faculty of Agronomy, University of Concepción, Av. Vicente Méndez, 595 Chillán, Chile; 5 National Key Laboratory of Crop Genetic Improvement, Huazhong Agricultural University, Wuhan, 430070 China; 6 School of Biological Sciences, The University of Western Australia, 35 Stirling Hwy, Crawley, Perth, 6009 Australia

**Keywords:** *Brassica*, interspecific hybridization, homoeologous exchange, hybrid stability, hybrid fertility, introgression

## Abstract

Interspecific hybridization is widespread in nature and can result in the formation of new hybrid species as well as the transfer of traits between species. However, the fate of newly formed hybrid lineages is relatively understudied. We undertook pairwise crossing between multiple genotypes of three *Brassica* allotetraploid species *Brassica juncea* (2*n* = AABB), *Brassica carinata* (2*n* = BBCC), and *Brassica napus* (2*n* = AACC) to generate AABC, BBAC, and CCAB interspecific hybrids and investigated chromosome inheritance and fertility in these hybrids and their self-pollinated progeny. Surprisingly, despite the presence of a complete diploid genome in all hybrids, hybrid fertility was very low. AABC and BBAC first generation (F_1_) hybrids both averaged ~16% pollen viability compared to 3.5% in CCAB hybrids: most CCAB hybrid flowers were male-sterile. AABC and CCAB F_1_ hybrid plants averaged 5.5 and 0.5 seeds per plant, respectively, and BBAC F_1_ hybrids ~56 seeds/plant. In the second generation (S_1_), all confirmed self-pollinated progeny resulting from CCAB hybrids were sterile, producing no self-pollinated seeds. Three AABC S_1_ hybrids putatively resulting from unreduced gametes produced 3, 14, and 182 seeds each, while other AABC S_1_ hybrids averaged 1.5 seeds/plant (0–8). BBAC S_1_ hybrids averaged 44 seeds/plant (range 0–403). We also observed strong bias towards retention rather than loss of the haploid genomes, suggesting that the subgenomes in the *Brassica* allotetraploids are already highly interdependent, such that loss of one subgenome is detrimental to fertility and viability. Our results suggest that relationships between subgenomes determine hybridization outcomes in these species.

**Supplementary Information:**

The online version contains supplementary material available at 10.1007/s10577-023-09733-9.

## Introduction

The *Brassica* genus is the most prominent in the Brassicaceae family and includes 39 species. Many of the species in this genus are cultivated for their edible roots, stems, leaves, buds, flowers, and seeds (oil and mustard) (Rakow 2004). The six most agriculturally important members of this group are described by the Triangle of U, which also established the chromosome number and genetic relationship between these cultivated species. The Triangle of U consists of three diploid and three allopolyploid species: diploid species *Brassica rapa* (*B. rapa*) (A genome, *n* = 10), *Brassica nigra* (*B. nigra*) (B genome, *n* = 8), and *Brassica oleracea* (*B. oleracea*) (C genome, *n* = 9) were determined to be the progenitors of the allopolyploid species *Brassica juncea* (*B. juncea*) (AB genomes, *n* = 18), *Brassica napus* (*B. napus*) (AC genomes, *n* = 19), and *Brassica carinata* (*B. carinata*) (BC genomes, *n* = 17) (U N 1935).

The ancestral relationship which exists between the *Brassica* A, B, and C genomes has been well elucidated (Attia and Röbbelen 1986; Lagercrantz and Lydiate 1996; Ge and Li 2007; Mason et al. 2010; Chalhoub et al. 2014). The A and C genomes have been shown to be more closely related to each other than to the B genome, with the *B. nigra* (B) lineage predicted to have diverged from the *B. rapa* and *B. oleracea* (A/C) lineage approximately 7.9 million years ago (Mya) followed by the separation of the *B. rapa* (A) and *B. oleracea* (C) lineages about 3.7 Mya (Inaba and Nishio 2002; Panjabi et al. 2008). Studies have also shown that the A and C genomes readily pair with each other. This has been demonstrated in synthetic AACC allotetraploids (Katche and Mason 2023), AC haploids (Nicolas et al. 2009), AAC and CCA triploids (Leflon et al. 2006), in trigenomic AABC, BBAC, and CCAB tetraploid hybrids (Mason et al. 2010), and in AABBCC allohexaploids (Gaebelein et al. 2019a, 2019b), with the highest frequencies observed in the absence of homologous chromosome pairing partners in the A and C genomes (allohaploids)(Nicolas et al. 2009). Although the B genome still shares a high degree of homoeology with the A and C genomes (Lagercrantz and Lydiate 1996; Perumal et al. 2020), A-B and B-C homoeologous pairing is less frequently observed (Mason et al. 2010; Chen et al. 2011; Navabi et al. 2011; Gaebelein and Mason 2018). The diploid A, B, and C genomes are also mesopolyploid, with a triplicated structure resulting from ancestral polyploidy events in the Brassiceae lineage (The Brassica rapa Genome Sequencing Project Consortium 2011; Parkin et al. 2014). These regions of secondary homoeology are also sufficient to induce chromosome pairing (autosyndesis) at low frequencies, e.g., in A genome (Armstrong and Keller 1981), B genome (Prakash 1973), and C genome (Armstrong and Keller 1982) haploids, and in AABC, BBAC and CCAB hybrids (Mason et al. 2010).

Allotetraploid × allotetraploid *Brassica* crosses can be readily carried out to produce hybrids containing all three *Brassica* A, B, and C genomes (FitzJohn et al. 2007; Katche et al. 2019). These allotetraploid species may be crossed in different combinations to produce AABC, BBAC, and CCAB hybrids which have been reported in several different experimental studies (Nelson et al. 2009; Mason et al. 2010; Navabi et al. 2010). Recently, we reported the fate of BBAC hybrids (Katche et al. 2021), but to date, very little is known about how AABC and CCAB hybrid lineages behave in subsequent generations following self-pollination. Each of the three allotetraploid parent species is self-compatible, so self-pollination success is expected to be a product primarily of the meiotic process and chromosome inheritance in these hybrids. In this study, we describe the production of three *Brassica* interspecific hybrid types; AABC, BBAC, and CCAB, by pairwise crossing of different self-compatible genotypes of the *Brassica* allotetraploid species *B. juncea*, *B. napus*, and *B. carinata*. We studied the chromosome inheritance, fertility, and stability of these hybrids following self-pollination, in order to shed light on possible pathways for natural hybridization and species formation.

## Materials and methods

### Experimental plant material and growth conditions

Three *Brassica* trigenomic hybrid populations AABC, BBAC and CCAB obtained by interspecific hybridization of *Brassica* allopolyploids were used for this study. Fertility for a subset of first generation (F_1_) hybrid plants and genotypes used in this study is presented in Mason et al. (2011). BBAC F_1_ and second generation (S_1_) hybrid types (derived from self-pollination of the F_1_ hybrids), along with information on the later generations of this cross, have already been described in detail (Katche et al. 2021) but are presented again here for the purposes of comparison between the three hybrid types. All AABC S_1_ and CCAB S_1_ data are newly presented, including fertility data (Supplementary File S1b-g), chromosome counts (Supplementary File S1f-g), and SNP genotyping information (Supplementary File S2a, b).

*Brassica juncea* genotype “JN9-04”, hereafter represented with the code “J1”, was crossed with five different genotypes of *Brassica napus* (Boomer, Monty_028DH, Surpass400_024DH, Trilogy, and Westar_010DH) from Canola Breeders Western Australia to obtain 133 AABC F_1_ hybrids. A total of 93 AABC S_1_ plants were produced by self-pollination of AABC F_1_ plants. One hundred and twenty four CCAB F_1_ plants from ten different cross combinations were obtained by crossing two genotypes of *B. carinata* (195923.3.2_01DH and 94024.2_02DH) hereafter referred to as “C1” and “C2”, with twelve genotypes of *B. napus* (Ag-Spectrum, Argyle, ATR Cobbler, AV-Sapphire, and Skipton from the Australian Grains Genebank (Mason et al. 2015); Ningyou7 from Huazhong Agricultural University; and Boomer, Surpass400_024DH, Monty_028DH, Trilogy, Westar_10DH, and Lynx_037DH from Canola Breeders Western Australia (Supplementary File S1a). Sixteen CCAB S_1_ plants were subsequently produced by self-pollination of CCAB F_1_ plants derived from crosses between the two aforementioned genotypes of *B. carinata* and three genotypes of B. *napus* (Surpass400_024DH, Boomer, and Trilogy). The parental *B. juncea* genotype J1 was crossed with two different *B. carinata* genotypes “C1” and “C2”, respectively, to generate two separate BBAC F_1_ hybrid genotypes: “J1C1” and “J1C2.” A total of sixty-two BBAC F_1_ plants were produced. Two hundred and twenty-seven BBAC S_1_ seeds produced from self-pollination of the J1C1 and J1C2 BBAC F_1_ genotypes were sown directly into the field at Huazhong Agricultural University, Wuhan, China. An additional 44 seeds were grown under glasshouse conditions at The University of Queensland, while seeds from all other BBAC F_1_ hybrids were germinated in potting mix and grown in pots in a controlled environment room (CER) at 18 °C/13 °C day/night with a 16 h photoperiod and light intensity of approximately 500 μmol m-2s-1. The three most fertile plants from each BBAC F_1_ plant, as measured by total self-pollinated seed produced, were selected to produce the next generation (one parent was selected from the growth room condition, five from the field condition).

### Fertility data collection

Total seed set was counted for all plants after encouraging self-pollination using micro-perforated plastic sleeves or paper bags to enclose racemes. Newly opened flowers were collected when plants started flowering and pollen stained with either fluorescein diacetate using the method detailed by Heslop-Harrison et al. (1984) (for the plants described in Mason et al. 2011, only pollen which fluoresced bright green were assumed to be viable) or with 1% acetic acid carmine stain (all other plants, plump and darkly stained pollen were assumed to be viable). At least 300 pollen grains were counted for each of two flowers per plant and the percentage pollen viability was recorded (Supplementary Files S1b-g). Plants were then bagged to encourage self-fertilization, and total seed counted after drying (Supplementary Files S1b-g).

### Plant hybrid status

As all parent species and genotypes in the cross combinations were self-compatible, several measures were taken to establish true hybrid status for progeny resulting from interspecific hybridization (Supplementary File S1b-g). For first generation hybrids (F_1_), plants were scored on one or multiple of the following: (1) plant morphology, (2) pollen morphology, (3) microsatellite marker inheritance of single alleles from both parents (see Mason et al. 2011 for details), or (4) whole-genome SNP array genotyping for parent allele inheritance. Plant morphological traits scored included leaf, stem and flower color, leaf margin serration, leaf lobe number and morphology, leaf and stem hairiness, and growth habit. The majority of F_1_ progeny sets resulting from specific parent combinations could be definitively characterized based on these phenotypic traits, with confirmation provided from microsatellite marker results (see Mason et al. 2011). Hybrid pollen morphology was also distinctive: pollen from true hybrid plants showed large size variation between pollen grains and more spherical appearance (as opposed to ovoid) for viable pollen in comparison to the pollen of the parent species (see Mason et al. 2011 for details). For second-generation progeny resulting from self-pollination of true F_1_ hybrid plants (S_1_ generation), only genome-wide SNP genotyping to determine if no unexpected alleles were present relative to the alleles predicted from the two parents was considered sufficient to distinguish between truly self-pollinated progeny and progeny resulting from foreign pollen contamination on to the maternal F_1_ plants.

### DNA extraction and marker-based genotyping for the AABC, BBAC, and CCAB hybrids

Leaf samples were collected in 2-ml micro-centrifuge tubes and stored at −20 °C until use. DNA was extracted using the “Microprep” method described in Fulton et al. (1995), except for 30 AABC plants which were extracted using the BioSprint 96 plant work station (Qiagen, Hilden, Germany). Sixty-one AABC hybrids, forty BBAC, and eighteen CCAB S_1_ hybrids were genotyped. All BBAC and CCAB hybrids and 31 AABC hybrids were genotyped using the Illumina Infinium 60K *Brassica* AC SNP array (Clarke et al. 2016). The remaining 30 AABC hybrid plants were genotyped using the Illumina Infinium 90K *Brassica* ABC SNP array. Hybridization protocols were performed according to the manufacturer’s instructions for all samples and the genotype data was visualized and exported using the Genome Studio v2.0.4 software (Illumina Inc., San Diego CA, USA).

A total of 52 149 SNPs were exported for the A and C genomes (Supplementary File S2a,b). Through BLAST alignment of the SNP probe sequences, A- and C-genome SNPs were located on the Damor-*bzh* v8 reference sequence (Bayer et al. 2017). SNP genotyping analysis followed established methodology (Mason et al. 2017). Briefly, for downstream analyses, SNPs which had a “no call” in > 10% of individuals within a haplotype block (*r*^2^ = 1) of called SNPs or which had a “call” in > 10% of individuals within a haplotype block (*r*^2^ = 1) of “no-call” SNPs were removed from all hybrid types, in addition to SNPs showing patterns of segregation inconsistent with determined genomic locations. For the AABC S_1_ hybrids, the A genome was filtered to retain only SNPs which were polymorphic between the parent *B. napus* and *B. juncea* genotypes in the A genome, while in the CCAB S_1_ hybrids, the C genome was filtered to retain only SNPs which were polymorphic between the parent *B. napus* and *B. carinata* genotypes for each hybrid combination. No allelic segregation was expected for the B and C genomes in AABC hybrids, the A and C genomes in BBAC hybrids, or for the A and B genomes in CCAB hybrids, and hence SNPs which were heterozygous within these genomes (indicative of multi-locus amplification/aspecific probe binding) were filtered out with respect to parental genotype controls. As well, SNPs which mapped to the A genome but which amplified in *B. carinata* (2*n* = BBCC) and SNPs which mapped to the C genome but which amplified in *B. juncea* (2*n* = AABB) were filtered out. S_1_ generation individuals for each of the AABC, BBAC, and CCAB hybrids were determined to be the product of unintentional cross-pollination when these individuals showed presence of alleles (in haplotype blocks, not individual SNPs which might result from errors) that were not present in either of the two parent genotypes.

### Molecular karyotyping

Molecular karyotyping was carried out in order to establish the number of chromosomes present in each of the A and C genomes (and B genome if genotyped) and the presence of non-homologous recombination events. Centromere locations used were initially mapped using the Darmor v.4.1 *B. napus* reference genome using the half-tetrad analysis (see Mason et al. (2016) for details) and subsequently relocated on the Darmor v. 8. 1 *B. napus* reference genome ((Bayer et al. 2017); see Katche et al. (2021) for reported positions). Presence of a centromere was taken as evidence for presence of a chromosome, regardless of other putative non-homologous translocation events present on that chromosome, as chromosome fragments without a centromere cannot be transmitted via mitosis or meiosis. In chromosome regions spanning at least 10 SNPs and > 1 Mbp, strings of no calls (NC) in the genotyping data were taken as indications of absence of this chromosome region, indicative of a non-homologous recombination event (Mason et al. 2017; Quezada-Martinez et al. 2022).

### Cytological chromosome counting

Root tips were collected in 0.04% 8-hydroxyquinoline solution and incubated for 2 h at room temperature, followed by another 2 h at 4 °C. Root tips were then transferred to Carnoy’s I solution (3:1 parts ethanol: acetic acid) and incubated for 24 h before being transferred to 70% ethanol for storage at −20 °C. The procedure for mitosis slide preparation from root tips was as reported by Mason et al. (2014a), using the DAPI as the fluorescent stain. Fluorescence images were captured using a Cool Snap HQ camera (Photometrics) on an Axioplan 2 microscope (Zeiss) and analyzed using the MetaVue (Universal Imaging).

### Statistical analysis and graphing

Genotypic effect of the trigenomic hybrids on total number of self-pollinated seeds and pollen viability was tested for using the one-way ANOVA in the base R version 4.0.2 (R_Core_Team 2022), followed by Tukey’s Honest Significant Differences test for post-hoc comparisons between hybrid types. The one-way ANOVA was also used to test for significant differences in genotype for the total number of self-pollinated seeds in the F_1_ and S_1_ generation of AABC, CCAB, and BBAC hybrids. Boxplots and stripcharts were also produced in the base package of R v.4.0.2. Pearson’s *χ*^2^ test statistic values and barcharts were produced using the Microsoft Office Excel (2019), and chromosome images were combined and annotated using the Microsoft Office Powerpoint (2019).

## Results

### True hybridity (F_1_) and self-pollinated (S_1_) status of AABC, CCAB, and BBAC hybrid plants

A total of 125 plants were obtained from five different cross combinations of *B. juncea* and *B. napus* (Supplementary File S1b). Of these, 123 plants were predicted to be true F_1_ hybrids (based on plant morphology, pollen morphology, and/or marker data), while two plants were derived from maternal self-pollination (Supplementary File S1b). Of the 123 F_1_ hybrid plants, two were found to derived from unreduced gametes produced by the maternal (*B. napus*) parent and hence to have a genome complement of 2*n* = AAABCC; these showed morphology similar to the maternal parent (Mason et al. 2011). The remaining hybrids were assumed to have 2*n* = AABC chromosome complements. To produce the BBAC hybrids, one genotype of *B. juncea* was crossed with two genotypes of *B. carinata*. A total of 62 plants were obtained, all of which were true F_1_ hybrids; one of these plants was phenotypically abnormal (and sterile) and was found to derive from an aneuploid (< *n*) *B. carinata* gamete (Mason et al. 2011) (Supplementary File S1c). To produce the CCAB hybrids, two genotypes of *B. carinata* and 12 different genotypes of *B. napus* were hybridized to produce 121 plants. Of these, 116 plants were true F_1_ hybrids and five plants were derived from self-pollination of the maternal parent (Supplementary File S1d).

AABC, CCAB, and BBAC F_1_ hybrid plants were self-pollinated in order to obtain S_1_ hybrid plants. A total of 93 AABC S_1_ hybrid plants were produced by self-pollinating F_1_ hybrids, with SNP genotyping data available for 78 S_1_ plants (Supplementary File S1e). Of the SNP-genotyped plants, 10 plants (13%) were true S_1_ generation resulting from self-pollination, and 68 plants (87%) resulted from unintentional cross-pollination. The status of the 15 plants which were not SNP-genotyped could not be determined. A total of 191 BBAC S_1_ plants were produced. From these, SNP genotyping information was only available for 38 plants. All 38 plants were true S_1_ self-pollinated progeny of their F_1_ hybrid parent based on SNP information (Supplementary File S1f; (Katche et al. 2021)). For the CCAB plants, 15 S_1_ hybrids were obtained, out of which 11 plants were SNP genotyped. Five of these 11 plants were true self-pollinated progeny, while six plants resulted from unintentional cross-pollination (Supplementary File S1g).

### Fertility of AABC, CCAB, and BBAC interspecific F_1_ hybrids

**Fig. 1:**
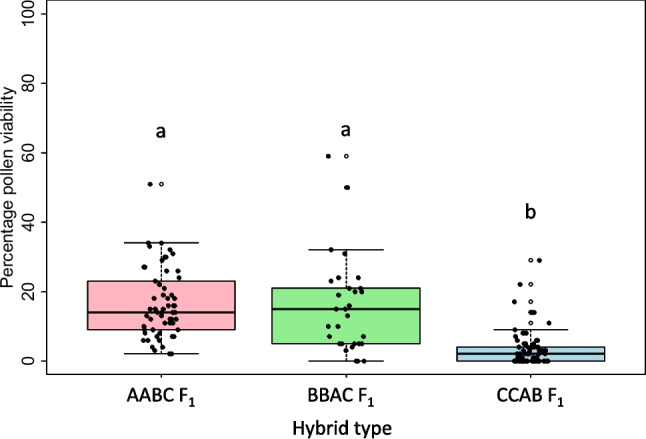
Pollen viability estimates in first generation (F_1_) interspecific *Brassica* hybrids with genome complements 2*n* = AABC, 2*n* = BBAC, and 2*n* = CCAB, derived from crosses between different genotypes of *B. napus*, *B. juncea*, and *B. carinata*. Different letters indicate significant differences between hybrid types (one-way ANOVA, *p* < 0.0001, followed by Tukey’s Honest Significant Differences, *p* < 0.0001)

**Fig. 2:**
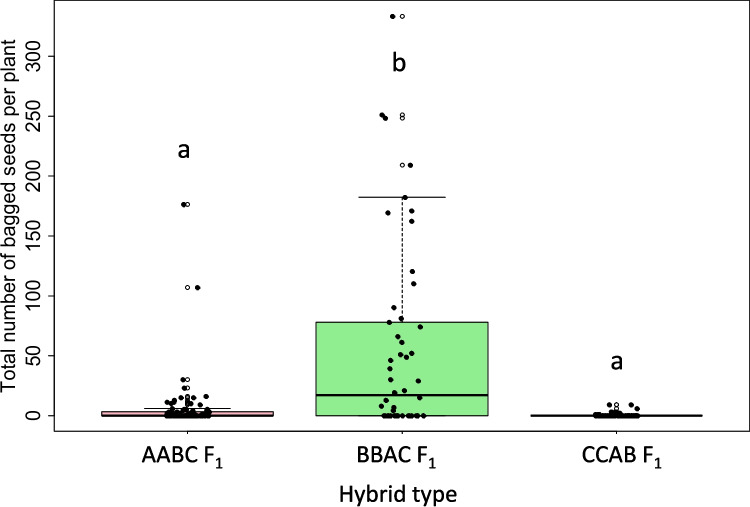
Bagged seed set per plant produced in first generation (F_1_) interspecific *Brassica* hybrids with genome complements 2*n* = AABC, 2*n* = BBAC, and 2n = CCAB, derived from crosses between different genotypes of *B. napus*, *B. juncea*, and *B. carinata*. Different letters indicate significant differences between hybrid types (one-way ANOVA, *p* < 0.0001, followed by Tukey’s Honest Significant Differences, *p* < 0.0001)

Fertility was assessed using seed set and estimated pollen viability as fertility measures in all true AABC, CCAB, and BBAC F_1_ hybrids (Supplementary File S1b-d). Pollen viability data was collected for 61 out of 121 AABC F_1_ lines and ranged from 2 to 51%, with an average of 16.6% (Fig. 1). The genotype combination *B. juncea* (JN9-04) × *B. napus* (Boomer) had the highest pollen viability while *B. juncea* (JN9-04) × *B. napus* (Monty_28DH) had the lowest. Bagged seed set was obtained for 91 out of 121 AABC F_1_ plants and ranged from 0 to 176 seeds per plant (Fig. 2), with an average seed fertility of 5.5 seeds per plant. Nearly half (43%) of the plants did not set any seed when bagged, and no significant difference between genotypes of different cross combinations for bagged seed set was observed (*p* < 0.05).

For the CCAB hybrids, a total of 116 F_1_ hybrid plants were produced. Pollen viability data was obtained for 73 true F_1_ plants and ranged from 0 to 29%, with an average of 3.5% (Fig. 1). Of all hybrid types, CCAB hybrids had the lowest percentage of viable pollen, with no viable pollen produced in 37% of plants. Number of seeds produced after bagging was obtained for 76 out of 116 hybrid plants, ranging from 0 to 9 seeds per plant, with an average of 0.5 seeds per plant (Fig. 2). Most (87%) of these plants did not produce any seed (Fig. 2), and no significant difference was detected between genotypes (*p* < 0.05).

For the BBAC hybrid population, a total of 62 true F_1_ hybrid plants were produced. Pollen viability data was obtained for 31 of these plants, which ranged from 0 to 59%, with an average of 16% (Fig. 1**)**. There was no significant difference between the two hybrid combinations J1C1 and J1C2 in terms of pollen viability (ANOVA, *p* = 0.164). Seed data in BBAC hybrids was obtained for 50 hybrid plants and ranged from 0 to 333 seeds per plant, with an average of 56 seeds per plant (Fig. 2). Nearly half (40%) of plants did not set seeds when bagged. No significant difference was observed between the two hybrid combinations J1C1 and J1C2 for number of self-pollinated seeds (ANOVA, *p* < 0.05).

The fertility of interspecific F_1_ hybrid types AABC, BBAC, and CCAB was compared for bagged seed set and pollen viability. BBAC hybrids produced the highest number of seeds per plant, followed by AABC hybrids and CCAB hybrids. Interestingly, similar proportions of AABC and BBAC F_1_ hybrids failed to produce any bagged seed, but fertile (at least one seed produced) BBAC F_1_ hybrids produced factorially more seeds per plant on average than fertile AABC F_1_ hybrids. CCAB F_1_ hybrids were more likely to be pollen-sterile, set no bagged seed, and to produce fewer seeds when fertile when compared to AABC and BBAC F_1_ hybrid types. Hybrid type was significantly associated with seed set and pollen viability (ANOVA, *p* = 8.67 × 10^−13^, *p* = 6.23 × 10^−15^, respectively, Tukey’s HSD *p* < 0.05). BBAC F_1_ hybrids were significantly different in the number of seed set compared to AABC and CCAB (Tukey’s HSD *p* < 0.05). BBAC hybrids were also significantly different in percentage pollen viability compared to CCAB but not AABC hybrids (Tukey’s HSD *p* < 0.05).

### Fertility of AABC, CCAB, and BBAC interspecific S_1_ hybrids

**Fig. 3:**
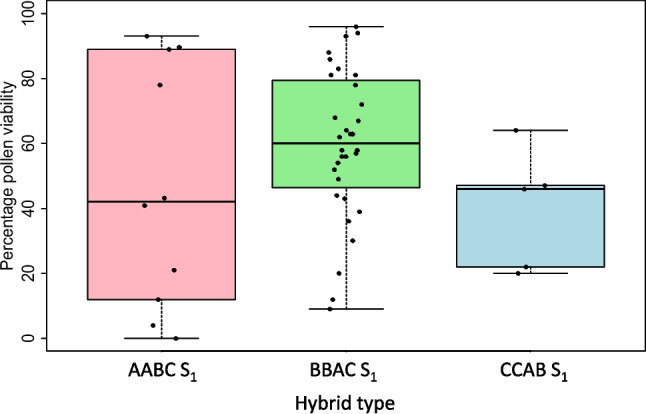
Pollen viability estimates in second generation (S_1_) interspecific *Brassica* hybrids derived from self-pollination of F_1_ hybrids with genome complements 2*n* = AABC, 2*n* = BBAC, and 2*n* = CCAB, derived from crosses between different genotypes of *B. napus*, *B. juncea*, and *B. carinata*. No significant differences were observed between hybrid types (one-way ANOVA, *p* > 0.05)

**Fig 4:**
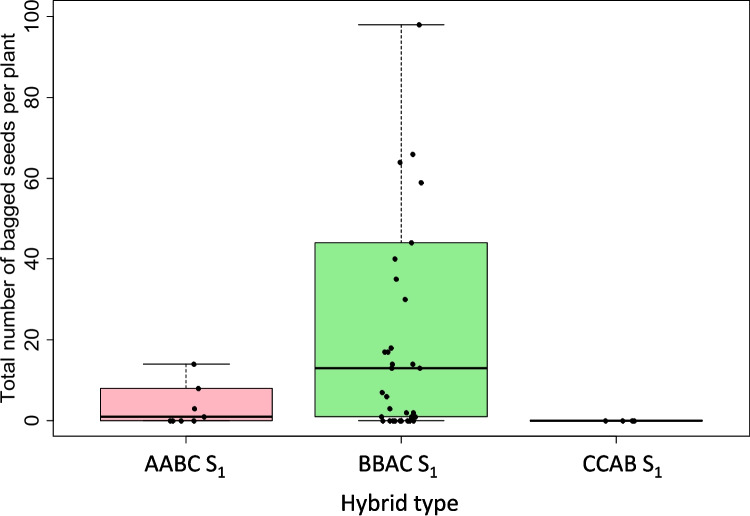
Bagged seed set per plant produced by second generation (S_1_) interspecific *Brassica* hybrids derived from self-pollination of F_1_ hybrids with genome complements 2*n* = AABC, 2*n* = BBAC, and 2*n* = CCAB, derived from crosses between different genotypes of *B. napus*, *B. juncea*, and *B. carinata*. No significant differences were observed between hybrid types (one-way ANOVA, *p* > 0.05)

AABC, CCAB, and BBAC F_1_ plants were bagged to encourage self-pollination to produce S_1_ plants. The fertility of true AABC, CCAB, and BBAC S_1_ hybrid plants was assessed by pollen viability and bagged seed production (Supplementary File S1e-g). For the 10 true AABC S_1_ hybrids (four genotype combinations, resulting from four different *B. napus* parent genotypes crossed with one *B. juncea* parent genotype), pollen viability ranged from 0 to 93% with an average of 47% (Fig. 3). Bagged seed set data was collected for 9/10 true AABC S_1_ hybrids and ranged from 0 to 182 seeds per plant, averaging 23 (Fig. 4).

Only five true CCAB S_1_ hybrid plants were obtained from self-pollination of F_1_ plants, all from different F_1_ hybrid plants resulting from a single-genotype combination “N1C2.” Pollen viability in the five plants ranged from 20 to 64%, with an average of 39% (Fig. 3). However, none of the CCAB S_1_ hybrids (only four were assessed) produced bagged seeds, indicating that these hybrids were completely sterile (Fig. 4).

A total of 38 true BBAC S_1_ plants were assessed for fertility. Pollen viability was assessed in 32 out of 38 true hybrids, and ranged from 9 to 96%, with an average of 59% (Fig. 3). Bagged seed set was assessed in 37 out of 38 true BBAC S_1_ hybrids and ranged from 0 to 403 per plant, with an average of 44 seeds/plant (Fig. 4). We observed significant differences in the number of bagged seeds produced per plant between the two different genotype cross combinations “J1C1” and “J1C2” (ANOVA, *p* = 0.05). A positive correlation was also observed between bagged seeds produced and pollen viability (*r* = +0.48).

The fertility of the three different S_1_ hybrid types was assessed using self-pollinated seed set and pollen viability in order to determine whether fertility is affected by AABC, CCAB, and BBAC S_1_ hybrid combinations or genotypes. Bagged seed set and pollen viability between AABC, BBAC, and CCAB S_1_ hybrids were not significantly different (ANOVA *p* > 0.05) (Fig. 3, Fig. 4). There was also no significant association between pollen viability or bagged seed set and genotype across all three hybrid types (ANOVA *p* > 0.05).

### Chromosome counts in the AABC, BBAC, and CCAB S_1_ hybrids

**Fig. 5:**
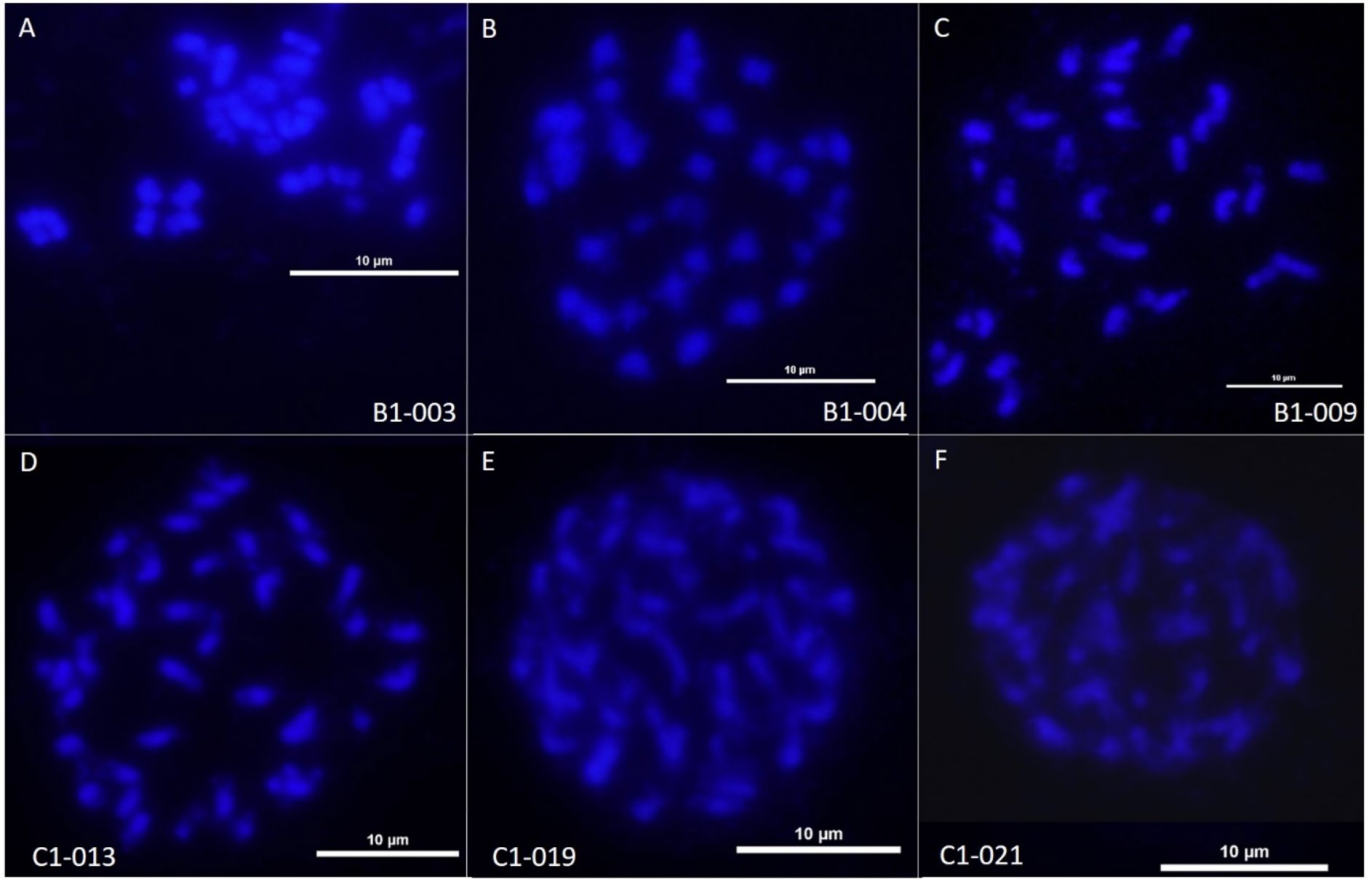
Mitotic chromosome spreads of self-pollinated progeny resulting from first generation hybrids between *Brassica juncea* and *B. carinata* (BBAC S_1_; **A**: B1-003, 2*n*~33 chromosomes; **B**: B1-004, 2*n*~36 chromosomes; and **C**: B1-009, 2n~34 chromosomes) and between *B. napus* and *B. carinata* (CCAB S_1_; **D**: C1-013, 2*n*~43 chromosomes; **E**: C1-019, 2*n*~44 chromosomes; and **F**: C1-021, 2*n*~42 chromosomes)

The chromosome numbers of 29 hybrid plants were counted in the S_1_ generation (Supplementary File S1f-g). For the CCAB S_1_ hybrids, 3/5 of the hybrid plants were analyzed, and had chromosome counts of 42, 43, and 44 chromosomes (Fig. 5). For BBAC S_1_ hybrids, chromosome counts were done for 24 plants. The chromosome number ranged from 29 to 36 with a mean of 33 and a mode of 35 (8 individuals, the same as the BBAC F_1_ parent). No chromosome information was obtained for true AABC S_1_ hybrids.

### Chromosome inheritance in AABC S_1_ hybrids

**Fig. 6:**
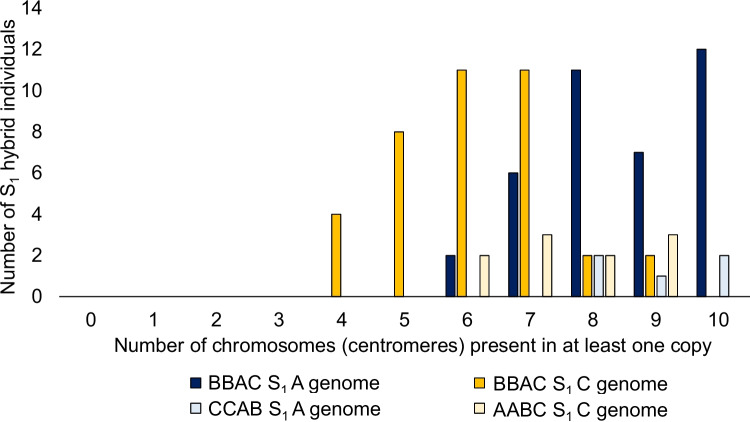
Inheritance of chromosomes belonging to initially haploid A and C genomes in *Brassica* interspecific hybrids with genome complements AABC, BBAC, and CCAB in the first generation, followed by one generation of self-pollination to produce S_1_ individuals. Dark blue and dark orange represent presence of A- and C-genome chromosomes in BBAC S_1_ individuals, respectively, pale blue represents presence of A-genome chromosomes in CCAB S_1_ hybrids and pale orange represents presence of C-genome chromosomes in AABC S_1_ hybrids. Presence or absence of chromosomes was assessed by presence of the centromeric region based on SNP array genotyping

A total of 10 true AABC S_1_ hybrid plants had SNP genotyping information: five plants had A, B, and C genome data, and five plants had A and C genome data. For inheritance of the haploid B and C genome chromosomes across all ten individuals, we expected to see a 1:2:1 segregation ratio of 0 copies:1 copy:2 copies of each B- or C-genome chromosome (as a result of self-pollination of a parent AABC F_1_ hybrid with one copy of each B- and C-genome chromosome). However, 0 C-genome chromosome copies were observed only 13% of the time, with statistically significant bias towards retention of C-genome chromosomes relative to the expected distribution (Pearson’s *χ*^2^ test, *p* = 0.011, Fig. 6). Upon closer inspection, seven out of ten individuals had one or two missing chromosomes plus one or two missing chromosome fragments (the latter indicative of non-homologous recombination events), while three individuals (A-01, A-02, and A3-001) inherited at least one copy of all C-genome chromosomes. B-genome data was only available for five individuals: A-01 and A-02 were not missing any B-genome chromosomes, A-65 was missing a copy of B1, A-71 was missing a copy of B7, and A-69 was missing three chromosomes completely (B4, B6, and B7) and was also missing most of chromosome B8, including the centromere.

Of the ten AABC S_1_ hybrids, seven showed no significant deviation from the expected 50% heterozygosity for loci segregating for parental *B. napus* and *B. juncea* alleles in the A genome (Pearson’s *χ*^2^ test, *p* > 0.05). Three individuals deviated significantly from expected chromosome segregation ratios (Pearson’s *χ*^2^ test, *p* < 0.0001), all showing higher heterozygosity than expected: 86%, 89%, and 87% heterozygosity in individuals A3-001, A-01, and A-02. Surprisingly, three individuals from the combination J1N2 with expected 50% heterozygosity (Pearson’s *χ*^2^ test *p* > 0.05) also demonstrated some bias towards retention of N2 alleles over J1 alleles (30%, 37%, and 32% homozygous for N2 alleles, as compared to expected 25%), although this only reached significance (*p* = 0.02) for individual A-69.

### Chromosome inheritance in BBAC S_1_ hybrids

A total of 38 BBAC S_1_ individuals were genotyped using the AC array: 20 from the genotype combination J1C1 and 18 from the genotype combination J1C2. Partial chromosome inheritance (a product of non-homologous recombination) was extremely common for the A and C genomes: on average, 40% of chromosomes showed this pattern (ranging from 5% of chromosomes for chromosome A08 to 68% of chromosomes for chromosome C9). Presence of a whole A- or C-genome chromosome was observed 54% of the time on average (ranging from 29% for chromosome C9 to 92% for chromosome A08), while complete absence of an A- or C-genome chromosome was observed only 6% of the time on average (chromosomes A03, A05, and C06 were always at least partially present, and chromosome C07 was completely lost most often, in 21% of individuals).

Inheritance of centromeric regions was used to assess presence of A- and C-genome chromosomes independent of non-homologous recombination events (partial chromosome presence) (Fig. 6). On average, based on centromere inheritance, A-genome chromosomes were lost 14% of the time and C-genome chromosomes were lost 32% of the time: both represent a significant deviation from the expected 25% retention of these chromosomes under a univalent segregation model (Pearson’s *χ*^2^ test, *p* <0.0001 and *p* = 0.003, respectively). Based on an expected 75% chance of each chromosome being present (following self-pollination of a parent F_1_ hybrid with one copy of each chromosome), chromosomes A04, A05, A06, and A08 were present more often than expected by chance (Pearson’s *χ*^2^ test, *p* = 0.039, *p* = 0.0050, *p* = 0.039, and *p* = 0.0015, respectively), and chromosomes C4 and C5 were lost more often than expected by chance (Pearson’s *χ*^2^ test, *p* = 0.00037 and *p* < 0.0001, respectively). Segregation of A- and C-chromosomes combined approximated the expected 25% inheritance of univalent chromosomes (Pearson’s *χ*^2^ test, *p* > 0.05).

### Chromosome inheritance in CCAB S_1_ hybrids

Only five true hybrid CCAB S_1_ individuals were identified, and all were genotyped with the AC array. C1-019 showed no partial or complete loss of chromosomes, but at least one such event was observed for each other CCAB S_1_ individual. Complete loss of chromosome A06 was observed in two individuals; no other chromosomes were lost completely although chromosomes A01 and A04 had undergone recombination events where only a small telomeric chromosome fraction was retained (in individuals C1-013 and C1-015, respectively). Large fractions of A-genome chromosomes were retained for events involving chromosomes A02 (in two individuals), A05, and A07.

Segregation of polymorphic loci in the C genome was surprisingly irregular: only one individual (C1-015) showed the expected 50% heterozygosity and approximately equal inheritance of alleles from the *B. napus* and *B. carinata* parent genotypes across all genomic loci (Pearson’s *χ*^2^ test, *p* > 0.05). One individual (C1-013) showed an excess of heterozygous loci (74%, Pearson’s *χ*^2^ test, *p* < 0.0001), while another individual (C1-019) showed an excess of homozygous loci (65%, Pearson’s *χ*^2^ test, *p* = 0.002). Individual C1-017 showed significant bias towards inheritance of *B. carinata* alleles over *B. napus* alleles (33% homozygous loci vs. 14%, Pearson’s *χ*^2^ test, *p* = 0.005), while three other individuals (C1-013, C1-019, and C1-021) showed the opposite trend, inheriting 18% vs. 8%, 40% vs. 25%, and 27% vs. 14% *B. napus* alleles relative to *B. carinata* alleles at homozygous loci (Pearson’s *χ*^2^ test, *p* = 0.04, *p* = 0.06, and *p* = 0.04, respectively).

## Discussion

In this study, we analyzed fertility and chromosome inheritance in *B. juncea* × *B. napus* (AABC), *B. juncea* × *B. carinata* (BBAC), and *B. napus* × *B. carinata* (CCAB) F_1_ interspecific hybrids and S_1_ generation plants resulting from self-pollination of the F_1_ hybrids. Although pollen viability in AABC and BBAC F_1_ hybrids was similar and higher than that of CCAB F_1_ hybrids (which were commonly male-sterile), only BBAC F_1_ hybrids produced substantial numbers of true S_1_ seeds. CCAB hybrids were all completely sterile by the S_1_ generation, while AABC S_1_hybrids were rescued from complete sterility by putative selection for unreduced gametes which transmitted complete sets of chromosomes from the F_1_ parent: three such individuals were identified and all produced at least a few seeds. Chromosome inheritance in BBAC S_1_ hybrids was biased towards retention of A-genome chromosomes and loss of C-genome chromosomes, and 40% of A- and C-genome chromosomes on average in these hybrids showed evidence of non-homologous recombination. Significant bias in allelic inheritance in the diploid A- and C-genomes of AABC S_1_ and CCAB S_1_ hybrids was also observed for several individuals, indicative of unreduced gamete involvement or other abnormal meiotic processes in both these hybrid types. As our experimental design mostly controlled for genotype-specific effects, our results suggest that fertility and viability of these hybrid lineages depend on genome structure, specifically on which genomes are present as haploid vs. diploid chromosome complements in the initial F_1_ hybrids.

Our results suggest that the fertility and hence viability of interspecific hybrid lineages depend strongly on chromosome inheritance patterns in the first generations following the interspecific hybridization event, which are in turn dependent on initial genome structure in the hybrid. Interspecific hybridization is ubiquitous in plants, animals, and microorganisms and has the potential to generate a large amount of genetic diversity over a short period of time (Grant and Grant 1994; Mallet 2005; Arnold and Martin 2009; Stukenbrock 2016). However, hybrid lineages often suffer from poor fertility, as we observed for these *Brassica* hybrid types. The poor fertility of hybrids can serve as a barrier preventing their maintenance as independent populations, thus hindering their long term speciation potentials (Charron et al. 2019). Indeed, in our study a CCAB (*B. napus* by *B. carinata*) hybrid lineage completely failed to establish, with universally low fertility despite the generation of many F_1_ hybrids from different genotype combinations. Different molecular mechanisms causing hybrid infertility exist, including genetic incompatibilities (nuclear and cytonuclear) and changes in genome architecture (ploidy number and chromosome rearrangements) (Rieseberg 2001; Maheshwari and Barbash 2011). Further physiological investigation of these hybrid types may be necessary to establish the exact mechanisms underlying the poor fertility observed.

We observed high levels of seed contamination resulting from inadvertent cross-pollination of the AABC and CCAB F_1_ hybrids, with the majority of S_1_ plants resulting from such inadvertent outcrossing, even under standard self-pollination conditions. Similar results were not observed for the more highly self-fertile BBAC S_1_ hybrids, for which almost all S_1_ progeny were pure. Contamination as a result of outcrossing to unknown parent may be a common fate of poorly self-fertile hybrids. In newly resynthesized *Brassica napus* hybrids, Katche et al. (2023) observed a 72% contamination rate using the SNP data analysis, which was attributed to possible outcrossing with established *B. napus* in the field where the hybrids were initially grown. Outcrossing of hybrids also often results in greater seed production than self-pollination (Schelfhout et al. 2008; Kumar et al. 2019). Possibly, inability to produce viable pollen, or failure of embryo development following self-pollination, results in strong selective pressure for otherwise rare cross-pollination events, resulting in high numbers of cross-pollinated progeny despite use of industry- and research-standard self-pollination methods in *Brassica*. Our results highlight the importance of using genome-wide markers to confirm parentage when working with highly infertile *Brassica* lines.

All our CCAB hybrids as well as most AABC hybrids were completely or nearly sterile in both the S_1_ and F_1_ generations, despite the presence of a complete diploid genome (CC or AA, respectively). Schelfhout et al. (2008) also observed substantially reduced F_1_ sterility in *B. napus* × *B. juncea* interspecific hybrid crosses in both directions. The fertility of these hybrids did not increase following self-pollination, as only one plant was able to survive self-pollination from the F_3_ to the F_4_ generation. Choudhary and Joshi (2001) also found *B. juncea* × *B. napus* AABC F_1_ hybrids to exhibit low fertility, which was attributed to chromosomal and genetic imbalance and/or cytoplasmic nuclear interactions. Similar results of low F_1_ pollen and seed viability in hybrids produced between *B. juncea* and *B carinata* (BBAC) were reported by Kumar et al. (2019). Our *B. juncea* by *B. carinata* hybrids showed higher fertility, but this fertility was found to be dependent on the presence of least one copy of each homoeologous region from each of the A or C genomes in later generations (Katche et al. 2021). Inheritance of complete haploid genome complements in our AABC S_1_ hybrids (as a probable result of unreduced gamete formation) also restored seed fertility. These results from our own and other studies suggest that a complete A or C genome resulting from the *Brassica* allotetraploid species *B. juncea*, *B. napus*, or *B. carinata* may no longer be sufficient for normal fertility in hybrids between these species. In support of this hypothesis, Pelé et al. (2017) also had trouble extracting the diploid A genome from *B. napus*, although a similar attempt to extract the A genome via intergeneric hybridization with *Isatis indigotica* followed by subgenome elimination was successful (Tu et al. 2010). Silencing and expression changes of subgenome-specific gene copies relative to their homoeologues have frequently been observed in *B. napus* (AACC) (Pan et al. 2019; Bird et al. 2021; Zhang et al. 2021; Wei et al. 2022); these changes may also negatively impact fertility after separation of the subgenomes in our interspecific hybrids.

In AABC, BBAC, and CCAB hybrids, frequent putative homoeologous exchanges involving the A and C genomes were detected. This high frequency of A/C pairing might have improved the chance of BBAC hybrids retaining at least one homoeologous gene copy from either of the A or C genomes, leading to improvement in seed fertility. In AABC, BBAC, and CCAB F_1_ interspecific hybrids, Mason et al. (2010) found that B/C associations were detected in the lowest frequency followed by A/B associations, while A/C associations were most frequently formed in all hybrid types, with an average of 7 A-C pairs per cell in BBAC F_1_ hybrids. In a report by Szadkowski et al. (2010), 30–47.5% A-C bivalents were observed in pollen mother cells of *B. napus* synthetics, with more than 10% of cells having more than three A-C bivalents. Similarly, high rates of recombination between the A and C genomes have been reported by other studies (Leflon et al. 2006; Szadkowski et al. 2010; Katche et al. 2021). These results can be explained by the genetic relationship between the A, B, and C genomes. Within the U’s Triangle species, the A and C genomes have been shown to be more closely related to each other than to the B and will also pair more readily during meiosis (Nicolas et al. 2007, 2009; Attia and Röbbelen 1986; Udall et al. 2005, Udall et al. 2005; Gaeta et al. 2007; Mason et al. 2010).

Three AABC S_1_ hybrids (A-01, A-02, and A3-001) and one CCAB S_1_ hybrid (C1-013) in our study showed (1) inheritance of all or almost all chromosomes from the haploid subgenome/s and (2) significantly elevated heterozygosity in the diploid (A or C) genome, consistent with involvement of unreduced gametes produced via a first division restitution-like mechanism. Unreduced gametes are known to be involved more frequently than expected by chance in crosses between species and to be produced at higher levels in interspecific hybrids (Ramsey and Schemske 1998; Mason and Pires 2015; Kreiner et al. 2017). In *Brassica*, unreduced gametes have been frequently observed in crosses between species and ploidy levels (Heyn 1977; Mason et al. 2011), as well as before in these specific hybrid types resulting from either cross-pollination events or microspore culture (Nelson et al. 2009; Mason et al. 2016). Unreduced gametes produced via a first division restitution-like mechanism (see (De Storme and Mason 2014) for review) inherit both homologous chromosomes from their parent, resulting in elevated heterozygosity, and inherit a copy of all univalent chromosomes. This appeared to confer a major advantage to seed fertility for these three unreduced gamete-derived AABC S_1_ hybrids, which produced 3, 14, and 182 seeds, respectively, while only two other AABC S_1_ hybrids produced seeds (1 and 8) and the other five AABC S_1_ hybrids were sterile. The CCAB S_1_ hybrid C1-013 did not produce any seed but also showed loss of most of chromosome A01, putatively due to a non-homologous recombination event. Another CCAB S_1_ hybrid (C1-019) may have been the result of an unreduced gamete produced via second division restitution (whereby both sister chromatids are inherited) or some other abnormal meiotic process: this individual inherited a copy of each A-genome chromosome and showed significant bias towards loss of heterozygosity. However, C1-013 and C1-019 had an estimated 43–44 chromosomes, which are low relative to the expectation (CCAB = 37 chromosomes for a 2*n* gamete). This is difficult to explain via a self-pollination event, but may suggest that unreduced ovules developed directly into embryos, which has previously been documented in *Brassica* (Eenink 1974). It is possible that the individuals with high heterozygosity and retention of at least one copy of each haploid genome chromosome may have resulted from the combination of two reduced (normal) gametes; the observation of other bias towards inheritance of alleles from specific parents in other individuals supports this hypothesis (strong selection for rare viable gamete/embryo combinations). However, the division of the AABC S_1_ hybrids into two groups, one group with retention of all haploid chromosomes and 86–89% heterozygosity in the diploid genome and one group with loss of chromosomes and/or chromosome fragments in the haploid genome and 41–54% heterozygosity, would seem to indicate two different meiotic processes were responsible for these two types of progeny. Further investigation is necessary to confirm this hypothesis, but our results suggest that involvement of unreduced gametes can boost success of interspecific hybridization events by increasing chromosome retention and hence fertility in otherwise infertile interspecific hybrids.

Significant bias towards presence of A-genome chromosomes and absence of C-genome chromosomes was observed in the BBAC S_1_ hybrids. This bias was specifically associated with preferential (centromere) inheritance of chromosomes A04, A05, A06, and A08 in the A genome and preferential loss of chromosomes C4 and C5 in the C genome. These tend to be chromosomes which are less likely to be involved in A-C pairing, for which the most common pairs are A01-C1, A02-C2, A03-C3, and A07-C6 (Mason et al. 2014a). However, this trend did not include chromosome C7, which after A08 is the second most unlikely chromosome to be involved in A-C pairing (Mason et al. 2014b), also C7 was only 11% recombined in our study, relative to 5% in A08 and an average of 40% across all chromosomes. C4 and C5 also had high recombination rates in our study, at 50 and 60%, respectively. Together, these results suggest that there is a bias towards retention of A-genome chromosomes that is reduced or removed by non-homologous pairing with the C genome. However, there is no evidence for a similar bias towards loss of individual C-genome chromosomes (e.g. C7): the preferential loss of chromosomes C4 and C5 appears to be linked to their frequent non-homologous recombination with chromosomes A04, A05, and A06 (Mason et al. 2014a). Although similar biases towards inheritance of A-genome chromosomes or chromosome fragments over C-genome chromosomes or chromosomes fragments have been previously observed in *Brassica* allohexaploids (Gaebelein et al. 2019b) and in later-generation BBAC hybrids (Katche et al. 2021), the reason for this bias is still unknown. Gene-expression-based subgenome dominance is present in *Brassica napus* for up to a third of genes (Wei et al. 2022), and the *Brassica* A and C genomes show excellent dosage compensation (replacement of function by the homoeologous gene copy; e.g., (Xiong et al. 2011; Samans et al. 2017; Gonzalo et al. 2019)), such that it seems unlikely that gene expression differences between subgenomes would have an immediate effect on gamete or embryo survival. However, in *Lolium × Festuca* interspecific hybrids, which show preferential loss of *Festuca* chromosomes, Majka et al. (2023) were able to attribute this preferential chromosome loss effect to subgenome-specific (*Lolium-*only) expression of kinetochore proteins controlling attachment of univalent chromosomes to microtubules, such that *Festuca* chromosomes were improperly attached and subsequently often lost as micronuclei following meiosis. Similar bias towards inheritance of *Allium roylei* over *A. cepa* chromosomes in *A. cepa × A. roylei* hybrids was attributed to female meiotic drive (Kopecký et al. 2022). Similar meiotic mechanisms may be operating in our BBAC S_1_ hybrids, but further investigation would be required to confirm or refute this hypothesis.

### Supplementary information

ESM 1 (XLSX 66 kb)

ESM 2 (XLSX 23817 kb)
